# The Modified Sparrow Search Algorithm with Brown Motion and Levy Flight Strategy for the Class Integration Test Order Generation Problem

**DOI:** 10.3390/biomimetics10040195

**Published:** 2025-03-21

**Authors:** Chongyang Jiao, Qinglei Zhou, Wenning Zhang, Chunyan Zhang

**Affiliations:** 1 Laboratory for Advanced Computing and Intelligence Engineering, Information Engineering University, Zhengzhou 450001, China; 2 Henan Information Engineering School, Zhengzhou Vocational College of Industrial Safety, Zhengzhou 450011, China; 3 School of Computer and Artificial Intelligence, Zhengzhou University, Zhengzhou 450001, China; ieqlzhou@zzu.edu.cn; 4 Software College, Zhongyuan University of Technology, Zhengzhou 450007, China; zhangwn@zut.edu.cn

**Keywords:** integration testing, test order, sparrow search algorithm, stubbing complexity, Brownian motion, Levy flight

## Abstract

Software testing identifies potential errors and defects in software. A crucial component of software testing is integration testing, and the generation of class integration test orders (CITOs) is a critical topic in integration testing. The research shows that search-based algorithms can solve this problem effectively. As a novel search-based algorithm, the sparrow search algorithm (SSA) is good at finding the optimal to optimization problems, but it has drawbacks like weak population variety later on and the tendency to easily fall into the local optimum. To overcome its shortcomings, a modified sparrow search algorithm (MSSA) is developed and applied to the CITO generation issue. The algorithm is initialized with a good point set strategy, which distributes the sparrows evenly in the solution space. Then, the discoverer learning strategy of Brownian motion is introduced and the Levy flight is utilized to renew the positions of the followers, which balances the global search and local search of the algorithm. Finally, the optimal solution is subjected to random wandering to increase the probability of the algorithm jumping out of the local optimum. Using the overall stubbing complexity as a fitness function to evaluate different class test sequences, experiments are conducted on open-source Java systems, and the experimental results demonstrate that the MSSA generates test orders with lower stubbing cost in a shorter time than other novel intelligent algorithms. The superiority of the proposed algorithm is verified by five evaluation indexes: the overall stubbing complexity, attribute complexity, method complexity, convergence speed, and running time. The MSSA has shown significant advantages over the BSSA in all aspects. Among the nine systems, the total overall stubbing complexity of the MSSA is 13.776% lower than that of the BSSA. Total time is reduced by 23.814 s.

## 1. Introduction

Software testing is a primary tool for ensuring software quality, detecting potential errors and threats in software, and enhancing the user experience [1]. Software testing consists of five stages: unit testing, integration testing, system testing, and acceptance testing [2]. Integration testing verifies that there are no errors in the connectivity between units. The CITO generation problem, i.e., how classes are integrated into a system in a certain order after they have been developed independently, is the key issue of integration testing. Different test orders require different stubbing cost. When testing a module, it is necessary to simulate the functionality of other modules called by the test module, where the simulated functional modules are called test stubs. For example, in the ATM system, which consists of 21 classes, if the class Bank is integrated first, and the class Bank depends on the classes Status, ATM, and Money, test stubs need to be designed to simulate the results of their calls to ensure that the class Bank is tested normally. A reasonable test order can diminish the overall stubbing complexity, the cost of testing and the time of testers. Therefore, before integration testing, how to arrange the order of class integration so that the test cost of this test order is minimized as much as possible is a hot spot in integration testing. The graph-based method [3,4] was initially used to solve the problem of CITO generation. Kung et al. [5] first developed a graph-based approach to minimize the number of required test stubs. Le Traon et al. [6] extracted frond edges from each strongly connected component and removed all of the incoming edges of the node with the maximal number of incoming or outgoing fronds. Hewett [7] presented an incremental strategy by including appropriate class candidates into the test order one by one. The CITO generation problem is an NP-complete problem for which it is difficult to gain an accurate solution and can be solved by treating the CITO generation problem as an optimization problem. Traditional optimization methods, such as gradient-based optimization method [8], often have limitations in the face of large-scale, multi-constrained and nonlinear engineering optimization problems. In recent years, search-based algorithms have been widely applied to solve engineering optimization problems [9]. These search-based algorithms, such as the genetic algorithm (GA) [10], the particle swarm optimization algorithm (PSO) [11], the simulated annealing algorithm (SA) [12], the aquila optimizer (AO) [13], smell agent optimization (SAO) [14], and the arithmetic optimization algorithm (AOA) [15]. Zhang et al. [16] applied PSO to solve the matter of generating CITO. Assunção et al. [17] put forward a modified non-dominated sorting genetic algorithm for solving the problem of integration and test order. The algorithm employs an elite strategy to retain the best individuals that appear in the evolutionary process of each generation, uses the crowding distance to maintain the diversity of the swarm, and searches for a balance between breadth seeking and depth seeking, and ultimately selects the CITO according to the criterion of Pareto optimal criteria. Guizzo et al. [18] employed a hyper-heuristic algorithm to provide multiple optional combinations of mutation and crossover operations for each evolutionary iteration, which ultimately resulted in a satisfactory order of class integration test. Mariani et al. [19] developed an offline hyper-heuristic algorithm called GEMOITO based on grammatical evolution (GE), which can automatically generate a multi-objective evolutionary algorithm to solve the CITO generation problem. Zhang et al. [20] presented a new fitness function to generate best solutions to better solve the CITO generation problems using GA. Borner and Paech [21] introduced the search method of SA and used attribute complexity, method complexity and test focus as optimization targets to determine an optimal integration testing order.

Xue and Shen [22] presented SSA, a novel and effective swarm intelligence optimization technique that draws inspiration from the feeding and anti-predator behaviors of sparrows. The SSA has the merits of simple principle, few arguments, fast convergence speed, easy implementation, etc., as well as being widely used in the fields of path planning [23], image classification [24], fault diagnosis [25], power load forecasting [26], etc. However, the SSA has the defect of premature convergence when solving complex optimization problems, especially in the late period of algorithm evolution, the population diversity is lessened, and it is prone to fall into local extremes. Currently, to improve the comprehensive performance of the SSA, researchers have developed a lot of improvement ways. Li and Wu [27] introduced the opposition-based learning strategy in the sparrow swarm initialization stage, and improved the population position update formula with cosine inertia weight to enhance the optimization accuracy. Ma et al. [28] proposed an enhanced multi-strategy SSA (EMSSA). The EMSSA uses three tactics to improve the SSA algorithm. One is to use adaptive-tent chaos mapping to enable high diversity and larger randomness in the initial group. The second is to introduce a weighted sine cosine algorithm (SCA) at the defender position update to avoid the algorithm falling into a local optimum. Third, the algorithm’s search ability is improved by perturbing the current optimal sparrow position using the triangle similarity principle. A learning SSA was presented by Ouyang et al. [29] to prevent the algorithm from reaching a local optimum. To strengthen the SSA’s global search capabilities, the method implements the lens reverse learning strategy during the discover stage. To lessen the blind search, an enhanced sine cosine technique is implemented at the follower stage. Yan et al. [30] raised an improved SSA, which subdivides the followers stage into two stages: global search and local search. The volatile helix factor and the advanced iterative search strategy are added in the two stages, respectively. Liang et al. [31] accelerated the convergence rate of the algorithm by adaptive weighting, and the reformative boundary processing strategy improved the convergence precision of the algorithm to some extent. Zhang and Ding [32] designed the chaotic SSA, which mainly uses logical mapping, adaptive hyper-parameters and mutation operators to reinforce the global search ability of SSA. A multi-strategy augmentation and quantum computing-based SSA was presented by Wu et al. [33]. The algorithm demonstrates its great durability and broad applicability by performing well in tackling many problems in various dimensions.

**Figure 1:**
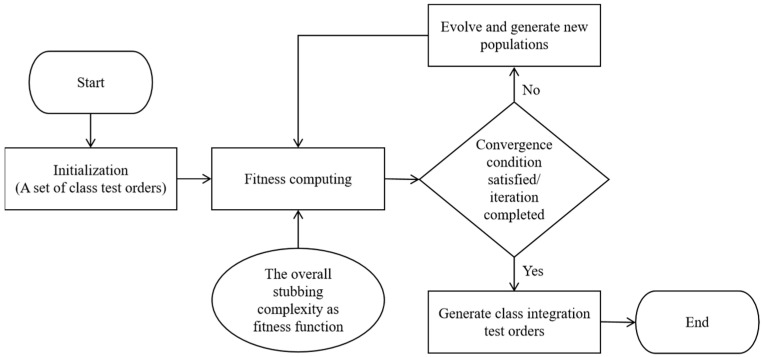
Flowchart of search-based CITO generation.

To reduce the testing cost of CITO generation, this paper adopts the search-based method to solve the CITO generation problem. The basic idea of the search-based method is as follows. Firstly, different initial populations are determined according to different program scales, and the CITO is mapped to individuals in the population. Then, the fitness function is established and the degree of the solution represented by each individual is judged by the fitness function; Finally, determine whether the algorithm satisfies the end condition or reaches the number of iterations. If not, execute the evolutionary operation operator to generate a new population, re-judge the degree of superiority of the solution represented by each individual through the fitness function, repeat the step until the end conditions are met and reflect the individual with the highest value of the fitness function as a test order to obtain the optimal test order. If satisfied, the generated class test sequence is the optimal class test order. The method framework is shown in Figure 1.

However, this approach can easily fall into local optimization when the number of classes is large, increasing the search time. SSA, as a new search-based algorithm, has the advantages of high convergence accuracy, fast convergence speed and strong robustness, and has been applied in many optimization problems. The CITO problem can be transformed into an optimization problem. This paper proposes a MSSA to generate the CITO problem. Firstly, a good point set strategy is utilized to initialize the population so that the sparrows are uniformly distributed in the solution space, which lays a good foundation for the evolution of the algorithm. Secondly, the Brownian motion model is applied to the discover update formula to obtain a larger foraging search range, and the Levy flight is used to update the follower’s position to improve the local domain search, so as to balance the exploratory and exploitative capacities of the algorithm. Then, a random wandering strategy is applied to the optimal solution to avoid the algorithm from falling into the local extremes. Finally, the overall stubbing complexity is designed as fitness function and the superiority of the algorithm is verified by nine open source software systems.

The main contributions of this paper are as follows:

- A MSSA is developed, which combines the initialization of the good point set strategy, the discoverer learning strategy of Brownian motion, the follower learning strategy of Levy flight, and the optimal solution random wandering strategy. To our best knowledge, this is the first time to introduce SSA to solve the CITO problem.
- The model of the MSSA to generate CITO is proposed, which includes four modules: the static analysis module of the software system to be tested, the class test order mapping module, the MSSA running module, and the optimal Sparrow mapping module.
- Experiments are conducted on nine open-source Java systems to demonstrate the superiority of the MSSA.

The rest of this paper is organized as follows. Section 2 provides the background required, including CITO generation issue and basic SSA. Section 3 presents our proposed method based on the MSSA for solving the CITO generation problem. The experiments and results analysis follow in Section 4. Section 5 discusses the limitations of our approach. Section 6 summarizes this work as well as points out the next research work.

## 2. Background

This section first introduces the CITO generation issue. Then, some necessary concepts are presented, including test stubs, attribute coupling, method coupling, attribute complexity, and method complexity. Finally, the BSSA is provided.

### 2.1. CITO Generation Issue

In object-oriented software systems, complex dependencies are created by message passing between classes [34]. To achieve different functions, each class has a different level of complexity and interaction with other classes. Important classes are more closely related to other classes, and if they have errors, it is easier for them to propagate the errors to the related classes and affect the function of the whole system. Therefore, in the integration testing, an important issue is to determine the integration of classes and the sequence of testing, which is called the CITO generation issue [35].

After determining the test order, software testers inevitably need to build test stubs. In an object-oriented program, the class being depended upon is called the service class, the class being served is called the client class, and the client class depends on the service class. In integration testing, if the service class is tested before the client class, there is no need to build test stubs because the service class has already been tested. When the client class is integrated first and the service class has not yet been integrated, testers need to build test stubs to simulate the functionality associated with the service class. Constructing suitable and correct test stubs is very costly, not only time-consuming and labor-intensive, but also requires a high level of professionalism on the part of the testers. Reducing the number of test stubs or the complexity of test stubs, and building the appropriate test stubs at the lowest possible cost can not only reduce the workload, but also improve the testing efficiency. The problem of CITO generation is to design an optimal order for integrating and testing classes. This order means that the number of test stubs created and the complexity of the test stubs are reduced to the minimum possible.

Suppose that a test system consists of n classes $C_{1},C_{2},\ldots ,C_{n}$, the CITO generation issue can be described as finding a class integration order that minimizes the overall cost of test stubs, i.e., to look for a class arrangement O{1,2,…,n} composed of n classes to minimize the overall cost of the test stubs that need to be constructed when testing according to the CITO $O(CO_{1},CO_{2},\ldots ,CO_{n})(n>1)$. The overall cost of test stub $stub_{\cos t}(CO_{1\ldots k})$ for test order $CO_{1\ldots k}=(CO_{1},CO_{2},\ldots ,CO_{k})$ is shown in Equation (1).

(1) $$stub_{\cos t}(CO_{1\ldots k})=\sum_{i=1}^{k}stub(CO_{i},CO_{i-1},\ldots ,CO_{1})$$

where $stub(CO_{i},CO_{i-1},\ldots ,CO_{1})$ is the cost of the test stub required to integrate class $CO_{i}$ into class collection $\left\{CO_{i-1},\ldots ,CO_{1}\right\}$. $stub_{\cos t}(CO_{1\ldots k})$ is the overall cost of test stub.

### 2.2. Background Concepts

**Definition** **1.** *Test stub. Given two classes i and j, class i depends on class j. When class i is tested in integration, but class j has not yet been tested in integration, class j is not available during the testing of class i. At this time, a simulation component is needed to simulate the behavior of class j, which is called a test stub.*

**Figure 2:**
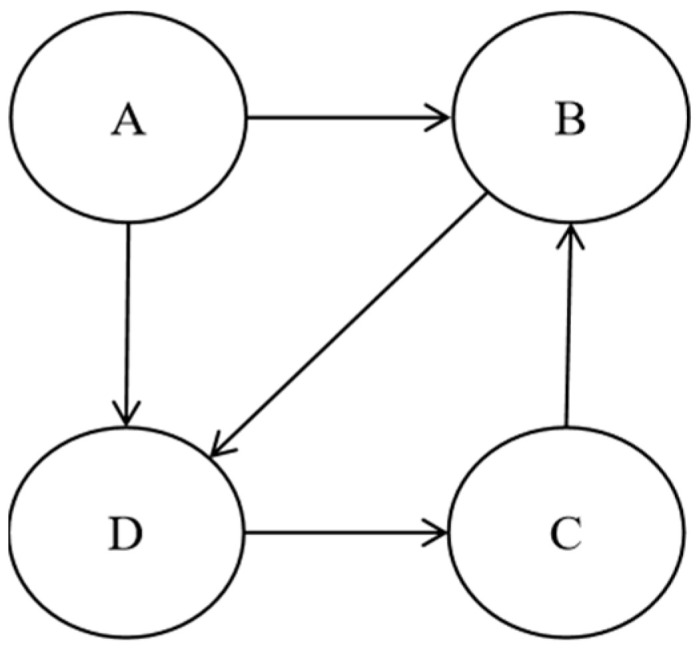
Example of a four-class program.

Test stub is generally categorized into generic stub and specific stub. Generic stubs are usually used to imitate the behavior of an entire class, which tends to add extra overhead, while specific stubs imitate a specific part of the class, such as a single method needed to use the class, which cannot be reused. Figure 2 gives a program that includes four classes, A, B, C, and D. The arrows indicate dependencies. Class A depends on classes B and D, class B depends on class D, class C depends on class B, and class D depends on class C. Table 1 lists the generic stubs and specific stubs required by the program.

Assume that the test order is (A,B,C,D). Since class A depends on classes B and D, and class B and class D need to be integrated after class A, a generic stub modeling class B and a generic stub modeling class D are needed for integration of class A. Class B depends on class D, and since a generic stub for class D has already been established, it does not need to be constructed again. Similarly, class C depends on class B, and since a generic stub for class B has already been built, there is no need to build it again. Class D depends on class C, which has been integrated and does not need additional test stubs. To sum up, for the test order (A,B,C,D), a total of two generic stubs need to be constructed, modeling class B and class D, respectively. Since class A depends on classes B and D, when integrating class A, it is necessary to build two specific stubs to simulate the method called by class A in class B and the method called by class A in class D, respectively. Similarly, class B depends on class D. Construct the specific stub of class D for class B. To summarize, for the (A,B,C,D) test order, three special stubs need to be constructed, i.e., class B for class A, class D for class A, and class D for class B.

**Definition** **2.** *Attribute coupling. If class i depends on class j, there are references to attributes declared locally by class j in the declaration lists of some methods of class i, or there are pointers to instances of class j in class i, then class i and class j have attribute coupling.*

**Definition** **3.** *Method coupling. If class i depends on class j, and class i calls methods of class j, then class i and class j have method coupling.*

**Definition** **4.** *Attribute complexity. If class i depends on class j and there exists attribute coupling between class i and class j, then the value of attribute dependency of class i on class j is called attribute complexity, denoted as $A\left(i,j\right)$.*

**Definition** **5.** *Method complexity. If class i depends on class j and there is method coupling between class i and class j, then the method dependency value of class i on class j is called method complexity, denoted as $M\left(i,j\right)$.*

### 2.3. The Basic Sparrow Search Algorithm

**Figure 3:**
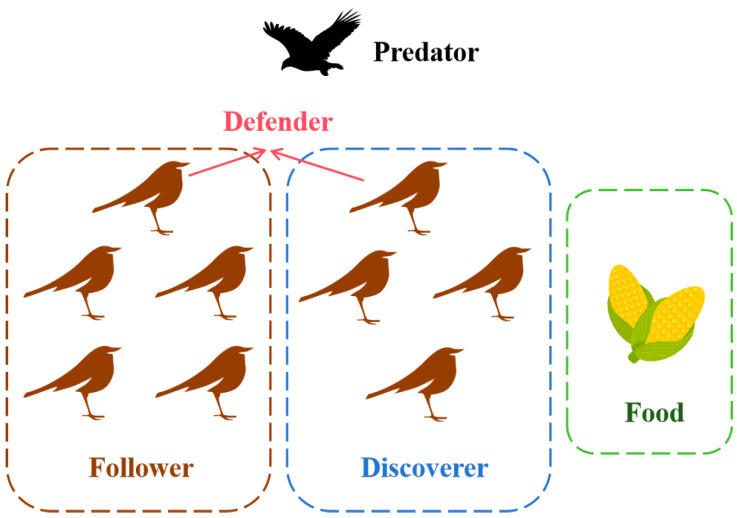
Model diagram of the BSSA.

Various types of creatures in nature can provide a source of ideas for human development, such as swarm intelligence algorithms, which simulate the group behavior of organisms in nature, and better adapt to the environment through the mutual cooperation between individuals, and then can constantly use the collective behavior for comprehensive optimization. The basic SSA (BSSA) is a swarm intelligence optimization algorithm that simulates the foraging mechanism of sparrows [22]. The model diagram of the BSSA is shown in Figure 3. The algorithm model contains three types of sparrow individuals, i.e., discoverers, followers and defenders [36]. The discoverer is responsible for finding the location with more abundant food in the whole search area and providing foraging direction for the followers. The follower will constantly monitor the discover, and as soon as the discover finds food, the follower will follow the discover to grab the food. Defenders are responsible for monitoring the feeding area, and when a predator is present around the foraging area, the defenders will immediately issue an early warning, and the entire population will immediately engage in anti-predator behavior and fly to a safe area.

Suppose that a population consists of N sparrows in a D-dimensional search space. The position of the i-th sparrow in the D-dimensional space is $X_{i}=[x_{i1},\ldots x_{id},\ldots x_{iD}],$ i=1,2,…,N. The fitness of the sparrow is denoted as $F=(f(X_{1}),f(X_{2}),\ldots ,f(X_{n}))$.

The positions of discoverers are modified by Equation (2).

(2) $$X_{i,j}^{t+1}=\left\{\begin{array}{ccc} X_{i,j}^{t}\cdot \exp(-\frac{i}{\alpha \cdot T_{\max}}) & if & R_{2}<ST\\ X_{i,j}^{t}+Q\cdot L & if & R_{2}\geq ST \end{array}\right.$$

where t is the current iteration, and $T_{\max}$ is the maximum number of iterations. $X_{i,j}$ represents the current position of the i-th sparrow in the j-th dimension. α∈(0,1] is a random number. Q indicates a random number obeying the standard normal distribution, and L means a 1×D matrix with each element is one. $R_{2}\in [0,1]$ and ST∈[0.5,1] are alarm value and safety threshold, respectively. When $R_{2}<ST$, there are no predators around the feeding area, and the discoverers can execute a thorough search mechanism. When $R_{2}\geq ST$, some individual have identified the predator and raised the alarm to other companions, and all sparrows should move rapidly to other safe places.

The positions of followers are updated by Equation (3).

(3) $$X_{i,j}^{t+1}=\left\{\begin{array}{ccc} Q\cdot \exp(\frac{X_{worst}-X_{i,j}^{t}}{i^{2}}) & if & i>N/2\\ X_{p}^{t+1}+\left|X_{i,j}^{t}-X_{P}^{t+1}\right|\cdot A^{+}\cdot L & if & i\leq N/2 \end{array}\right.$$

where $X_{p}$ represents the current optimal location of discoverers. $X_{worst}$ is the worst location of sparrows. The parameter A is a 1×D matrix in which each element is assigned 1 or −1 randomly, and $A^{+}=A^{T}{\left(AA^{T}\right)}^{-1}$. When i>N/2, the follower i does not obtain any food, is hungry, and needs to move to gain food. When i≤N/2, the follower i will fly around the $X_{p}$ for foraging.

To defend against predators, 10 to 20 percent of the sparrows from population are picked up as defenders randomly, and their positions are calculated as shown in Equation (4).

(4) $$X_{i,j}^{t+1}=\left\{\begin{array}{ccc} X_{best}^{t}+\beta \cdot \left|X_{i,j}^{t}-X_{best}^{t}\right| & if & f_{i}>f_{g}\\ X_{i,j}^{t}+K\cdot (\frac{X_{i,j}^{t}-X_{worst}^{t}}{(f_{i}-f_{w})+\varepsilon }) & if & f_{i}=f_{g} \end{array}\right.$$

where $X_{best}$ expresses the current best location of the population. β indicates the step-size control parameter that follows a normal distribution with a mean value of zero and a variance of one. K∈[−1,1] represents a random number. ε is an arbitrarily small constant to avoid the denominator from zero. $f_{i}$ is the fitness value of i-th sparrow, while $f_{g}$ and $f_{w}$ are the best and worst fitness values of the current sparrow population, respectively. $f_{i}>f_{f}$ means that the sparrow is at the edge of the population and is vulnerable to predators. $f_{i}=f_{g}$ demonstrates that the sparrows in the center of the population are aware of the danger and need to fly to other sparrows to avoid being attacked by predators.

**Figure 4:**
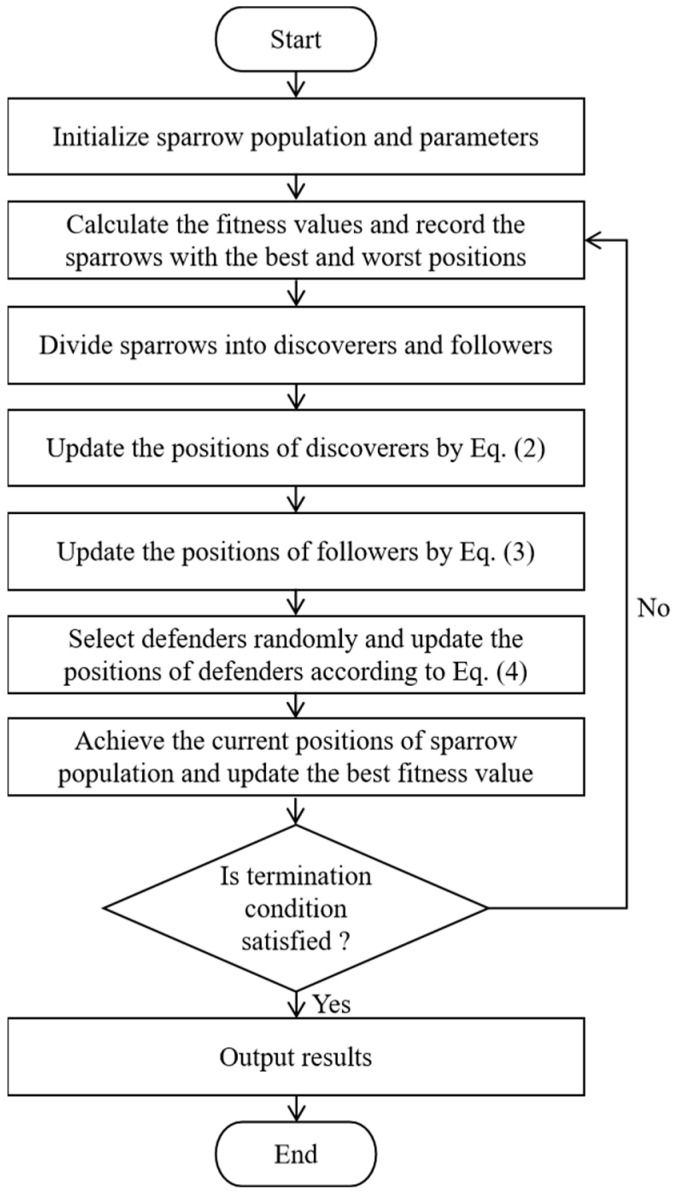
The flowchart of the BSSA.

Figure 4 illustrates the flowchart of the BSSA, while its pseudo-code is provided in Algorithm 1.
**Algorithm 1:** Pseudo-code of the BSSA
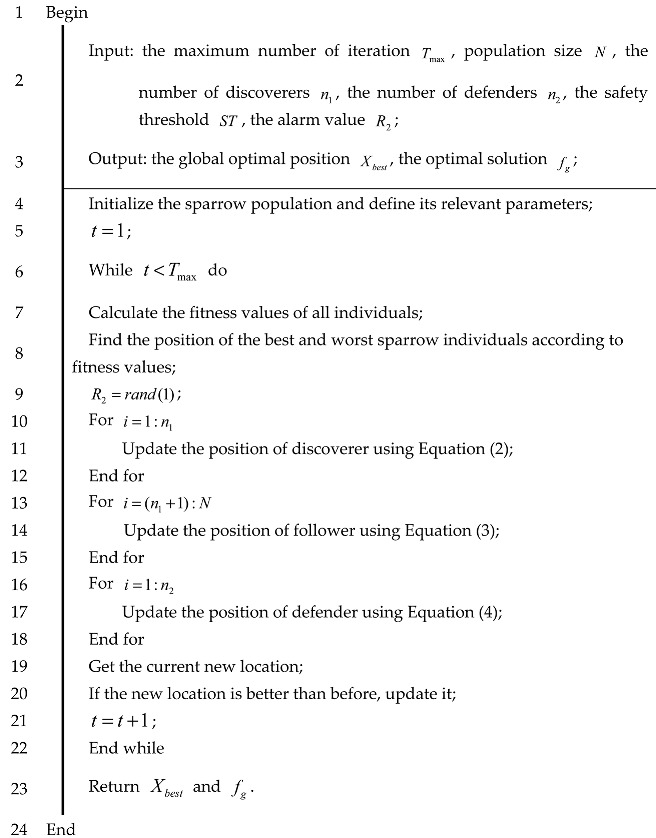


## 3. Methodology

In this section, the proposed MSSA is elaborated and applied to the CITO generation problem. In Section 3.1, the initialization strategy based on good point set is proposed. The discoverer learning strategy based on Brownian motion and the follower learning strategy based on Levy flight are presented in Section 3.2. In Section 3.3, the optimal solution random wandering strategy is developed. The fitness function is designed in Section 3.4. Section 3.5 details the model for generating CITO using the MSSA.

### 3.1. The Initialization Strategy Based on Good Point Set

The BSSA initialize the population in a random way, which leads to the randomness of the distribution of the population, and easily leads to the SSA falling into the local optimum. Due to the stochastic function, too many sparrows are generated at the edge of the search space, resulting in loss of population diversity and insufficient stability of the algorithm. To address this problem, this paper uses the good point set technique. The sparrow swarm is initialized by the good point set strategy, so that the initial sparrow population can be more evenly distributed in the search space to improve the convergence speed of the algorithm.

Two mathematicians, Hua Luogeng and Wang Yuan, came up with the approach of good point set. Selecting points with a good point set is much less biased than randomly selecting points [37]. The principle of a good point set is as follows.

Let $G_{m}$ be the unit cube in m dimensional Euclidean space, that is $x\in G_{m},\;x=(x_{1},x_{2},\ldots ,x_{m})$, where $0\leq x_{i}\leq 1,\;i=1,2,\ldots ,m.$ Let $r\in G_{m}$, the deviation $\varphi_{\left(n\right)}$ of the shape of $P_{n}(k)=(\{r_{1}^{\left(n\right)}\cdot k\},\{r_{2}^{\left(n\right)}\cdot k\},\ldots ,\{r_{m}^{\left(n\right)}\cdot k\})$, 1≤k≤n, satisfy $\varphi (n)=C(r,\varepsilon )n^{-1+\varepsilon }$, where C(r,ε) is a constant that solely affects r,ε(ε>0), then $P_{n}(k)$ is called the set of good points, and r is called a good point.

Equation (5) represents mapping the good points on $G_{m}$ to the sparrow search space.

(5) $$X_{i,j}=\{r_{j}^{\left(i\right)}\cdot k\}\cdot (ub_{j}-lb_{j})+lb_{j}$$

where $X_{i,j}$ is the position information of the i-th sparrow in the j dimension. $ub_{j}$ and $lb_{j}$ are the upper and lower bounds of the j dimension, respectively.

Algorithm 2 gives the pseudo-code to initialize sparrow population with good point set strategy.
**Algorithm 2:** Pseudo-code for initializing population with a good point set
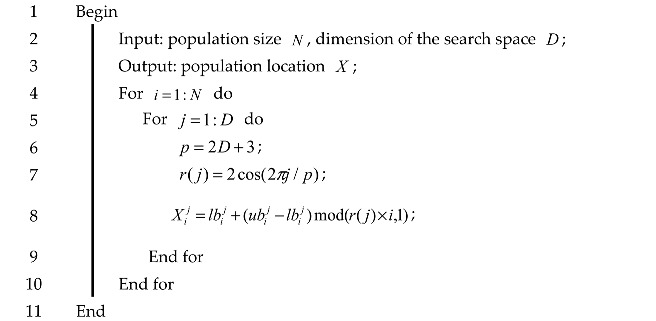


**Figure 5:**
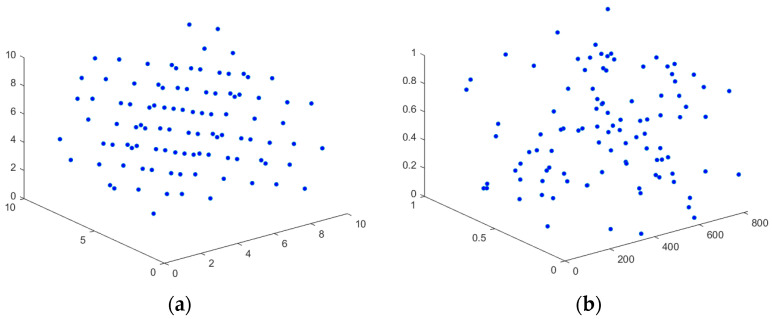
The distribution of individuals. (**a**) Good point set initialization. (**b**) Random initialization.

To verify the superiority of the good point set initialization strategy, set the population size to 100, and initialize the sparrow population in two-dimensional space. Figure 5a,b show the effect diagrams of adopting the good point set initialization and the traditional random initialization, respectively. It can be seen intuitively from Figure 5 that the individuals initialized by the random approach appear the phenomenon of individual aggregation, which can not effectively traverse the whole search space, while the sparrow population initialized by the strategy of the good point set is more uniformly distributed, which can further improve the convergence rate of the algorithm and avoid falling into the local optimum.

### 3.2. Brownian Motion and the Levy Flight Strategy

#### 3.2.1. Brownian Motion and Levy Flight

Brownian motion (BM) refers to the ceaseless, never-ending irregular motion of particles suspended in a liquid or gas, which plays a vital role in the scientific field [38]. BM is a stochastic process whose step size depends on a Gaussian distributed probability function with mean zero and variance one, which is able to cover the search space with a more uniform and controllable step size. The probability density function of the BM at the point x is shown in Equation (6).

(6) $$f_{B}(x;\mu ,\sigma )=\frac{1}{\sqrt{2\pi \sigma^{2}}}\exp(-\frac{{\left(x-\mu \right)}^{2}}{2\sigma^{2}})=\frac{1}{\sqrt{2\pi }}\exp(-\frac{x^{2}}{2})$$

where the step size of the particle motion is determined by the mean 0, i.e., μ=0, and the variance 1, i.e., σ=1.

Levy flight (LF) is a stochastic process invented by French mathematician Paul Levy in the 20th century. During Levy flight, the step size in each step obeys the Levy distribution, which has the characteristics of a heavy-tailed distribution, i.e., higher tail probabilities [39]. The Levy flight typically moves at smaller steps, but occasionally larger jumps can occur [40]. The probability density of the stabilization process of the Levy flight is defined as shown in Equation (7).

(7) $$L_{\alpha ,\gamma }(x;\alpha ,\gamma )=\frac{1}{\pi }\int_{0}^{\infty }e^{-\gamma p^{\alpha }}\cos(px)dp$$

where r is the scale factor r>0, and α represents the distribution exponent, 0<α≤2.

**Figure 6:**
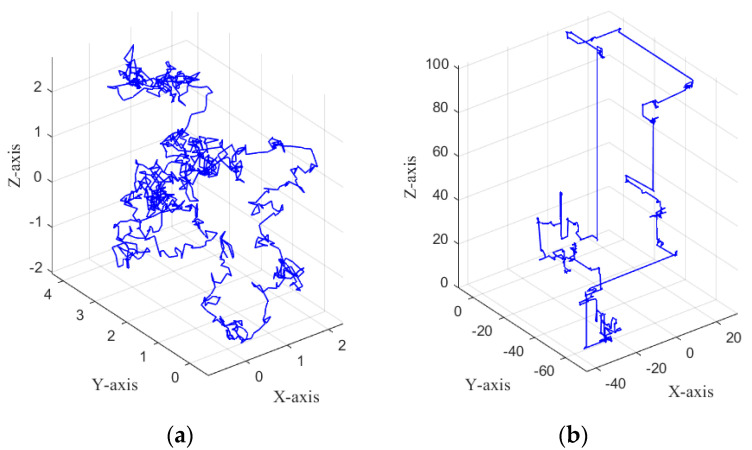
BM and LF in motion trajectory. (**a**) Three-dimensional trajectory of BM. (**b**) Three-dimensional trajectory of LF.

The trajectory diagrams of BM and Levy flight are shown in Figure 6.

As can be seen from Figure 6, the BM model has a wider coverage of the motion trajectory and a more uniform step size in the control area. LF consists of a mixture of many short steps and occasional long steps. This combination allows for local exploration and global exploration.

#### 3.2.2. The Discoverer Learning Strategy Based on Brownian Motion

The discoverer should be given a larger foraging search range than the followers because the BSSA primarily depends on the discoverer to locate food. The discoverer is also in charge of finding food for the whole population and informing all of the followers about foraging directions. From the Equation (2) in the BSSA, when $R_{2}<ST$, it indicates that the foraging habitat is free of predators, allowing the discoverer to conduct a thorough search. If $R_{2}\geq ST$, the current environment is dangerous, and some sparrows have already detected the predator and alerted the other individuals. At this time, all sparrows need to fly quickly to other safe places for finding food. In the BSSA, when the warning value is reached, the discoverer adopts the uniformly distributed random number step size factor to just move Q units, where Q is a random number conforming to the normal distribution, which leads to the problem of excessive randomness. If the step size of the movement is relatively too long, the discoverer will exceed the search boundary frequently, leaving a large number of sparrows in the entire swarm at the boundary and losing the original information. The BM model has a wider coverage and a more uniform step size, which can explore farther regions. To resolve the issue that the search strategy of the discoverer is too single and random, this paper introduces the BM step-size strategy to change the discoverer’s location, and the way it changes is shown in Equation (8).

(8) $$X_{i,j}^{t+1}=\left\{\begin{array}{ccc} X_{i,j}^{t}\cdot \exp(-\frac{i}{\alpha \cdot T_{\max}}) & if & R_{2}<ST\\ X_{i,j}^{t}+PR(R_{B}(X_{best}^{t}-R_{B}X_{i,j}^{t})) & if & R_{2}\geq ST \end{array}\right.$$

where R is the random number in the interval (0,1) and $R_{B}$ represents the BM step, P=1/2. $R_{2}\in [0,1]$ and ST∈[0.5,1].

BM is suitable for long-distance random search, while Levy flight with smaller step size can efficiently and deeply search nearby ground neighborhoods, and with longer step size can explore other areas. To better balance the exploration and exploitation capabilities of the algorithm, this paper combines BM with the Levy flight model.

#### 3.2.3. The Follower Learning Strategy Based on Levy Flight

According to the BSSA model, it can be seen that the follower constantly monitoring the discoverer. When the follower perceives that the discoverer has searched for a better food area, they will fly to the vicinity of the discoverer in large numbers, which leads to too high population density near the discoverer, and thus the algorithm is prone to fall into local extreme values. Levy flight is a special kind of random roaming, whose step size follows Levy distribution, which can generate large perturbations with a certain probability, thus enhancing the global search ability of the algorithm. The model can produce a variety of step sizes, and has certain ergodicity and randomness. Through high-frequency close detection and low-frequency remote detection, Levy flight is able to balance local development and global exploration in the search space, effectively reducing the risk of algorithms falling into local optimal. To avoid the algorithm falling into local extreme values, this paper proposes a follower learning strategy based on Levy flight, which improves the evolutionary formula of the follower by means of the Levy flight model. In this way, not only the search scope of the algorithm is expanded, but also the algorithm is avoided to fall into the local optimal. The description of follower position update based on Levy flight is shown in Equation (9).

(9) $$X_{i,j}^{t+1}=\left\{\begin{array}{c} Q\cdot \exp(\frac{X_{worst}-X_{i,j}^{t}}{i^{2}})\begin{array}{ccc} & if & i>N/2 \end{array}\\ X_{p}^{t+1}+\left|X_{i,j}^{t}-X_{P}^{t+1}\right|\cdot \frac{\theta }{{\left|\omega \right|}^{1/\gamma }}\begin{array}{ccc} & if & i\leq N/2 \end{array} \end{array}\right.$$

where

(10) $$\theta \sim N(0,\sigma_{\mu }^{2}),\;\omega \sim N(0,\sigma_{\nu }^{2})$$

where $\sigma_{\nu }=1$.

(11) $$\sigma_{\mu }={\left\{\frac{\Gamma (1+\gamma )\sin(\pi \gamma /2)}{\Gamma [(1+\gamma )/2]\gamma 2^{(\gamma -1)/2}}\right\}}^{1/\gamma }$$

where γ=1.5, Γ(x)=(x−1)!, x belongs to the set of natural numbers.

The feature of small-step tracking of the Levy flight model can help the algorithm to augment the local domain search, so that the algorithm can fully explore in a more optimal region and improve the accuracy of the search. Moreover, the features of long jumps of the model can perturb the population position and the stable path of the algorithm, which help the algorithm to achieve local search in a wider area, and help the algorithm to jump out of the local optimum and increase the diversity of the solution. Therefore, the follower learning strategy based on Levy flight enriches the diversity of population positions, guides other sparrows to find better positions, and effectively enhances the running efficiency.

### 3.3. The Optimal Solution Random Wandering Strategy

Although the discoverer learning strategy based on BM and the follower learning strategy based on LF can maintain the exploration and development capacity of the algorithm, it may still experience premature convergence. In the late iteration of the BSSA, the sparrows gradually approach the optimal individual, and the diversity of the group decreases, which can easily fall into the local optimum. To avoid this problem, this paper proposes the optimal solution random wandering strategy, which performs Cauchy random wandering, differential evolution random wandering, and Levy flight random wandering for the individual with the best fitness value in the group to increase the possibility of the algorithm to jump out of the local optimum.

The Cauchy distribution shows a tendency of protruding in the middle, flattening at both ends and descending gently, and this perturbation step size can make the population change more evenly, which improves the ability to jump out of the local optimal solution and at the same time ensures the convergence ability of the algorithm. The method of Cauchy random wandering is defined as shown in Equation (12).

(12) $${X_{best}^{d}}^{\prime }=X_{best}^{d}+F(c;c_{0},\mu )\times (X_{r1}^{d}-X_{r2}^{d})$$

(13) $$F(c;c_{0},\mu )=0.5+\frac{1}{\pi }\arctan(\frac{c-c_{0}}{\mu })$$

where $X_{best}^{d}$ is the d-dimensional value of the globally optimal sparrow found so far, and ${X_{best}^{d}}^{\prime }$ is the optimal sparrow after performing Cauchy random wandering. r1 and r2 are randomly selected from the entire sparrow group, and $r_{1}\neq r_{2}$. μ represents the scale parameter and μ>0. c is the random position and $c_{0}$ is the location parameter of the peak value of Cauchy distribution. $F(c;c_{0},\mu )$ is a random number produced by the Cauchy distribution function with μ=1 and $c_{0}=0$, and its calculation formula is shown in Equation (13).

Differential evolution algorithm is iterative through the variation, crossover and selection operations of the population, which makes the algorithm converge to the global optimum, and has the advantages of simplicity and efficiency [41]. To boost the convergence rate of the algorithm, the differential evolution random wandering strategy proposed is defined as shown in Equation (14).

(14) $${X_{best}^{d}}^{\prime }=X_{best}^{d}+rand\times (X_{r1}^{d}-X_{r2}^{d})+rand\times (X_{r3}^{d}-X_{r4}^{d})$$

where rand is a random number at [0,1]. r1, r2, r3, and r4 are randomly selected from the entire sparrow population, and $r_{1}\neq r_{2}\neq r_{3}\neq r_{4}$.

The Levy flight random wandering strategy is shown in Equation (15).

(15) $${X_{best}^{d}}^{\prime }=X_{best}^{d}+\beta_{1}\times L(\beta_{2})\times (X_{r1}^{d}-X_{r2}^{d})$$

where r1 and r2 are randomly selected from the whole population, and r1≠r2. μ and v denoted as the normal distribution.

(16) $$\beta_{1}=0.01+\frac{1}{1+e^{(40\frac{t}{T_{\max}})-20}}$$

(17) $$L(\beta_{2})=\frac{\mu }{|v{|}^{1/\beta_{2}}}$$

(18) $$\mu \sim N(0,\sigma_{\mu }^{2}),\;v\sim N(0,\sigma_{\nu }^{2})$$

where $\sigma_{v}=1$, $\sigma_{\mu }$ is shown in Equation (11).

The specific steps of the optimal solution random wandering strategy are shown in Algorithm 3.
**Algorithm 3:** Pseudo-code for random wandering optimal solution
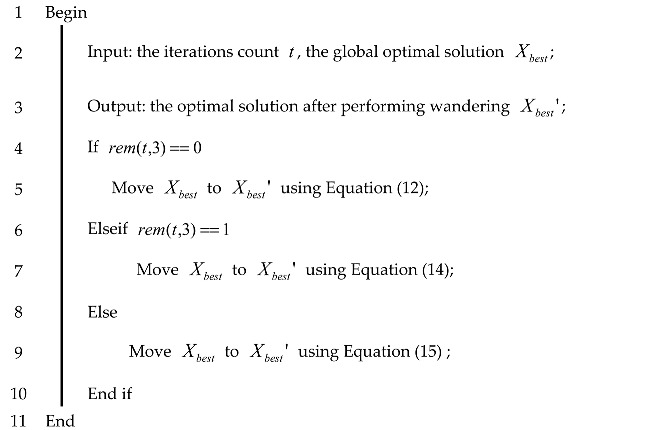


The specific process of the strategy for optimal solution random wandering is as follows.

Step 1: Record the current number of iterations t;

Step 2: If the number of iterations t can be divided by 3, the location of the best sparrow is wandered according to Equation (12);

Step 3: If the number of iterations t is divisible by 3 and the remainder is 1, the best sparrow wanders according to Equation (14);

Step 4: If the number of iterations t is divided by 3 and the remainder is 2, the best sparrow wanders according to Equation (15).

In the process of the MSSA search, along with the number of iterations, the introduction of three optimal solution random wandering strategies can better avoid the algorithm from falling into local extremes. Among them, Cauchy wandering allows the step length of the best sparrow move at a constant speed, Levy flight wandering can make the step length of the optimal sparrow increase, and Differential evolution wandering can reduce the step length of the optimal sparrow. The three random wandering strategies cooperate with each other to avoid premature convergence of the algorithm.

### 3.4. Construction of the Fitness Function

In object-oriented software systems, due to the complex dependencies between classes, one needs to spend lots of effort to establish test stubs to simulate the service required by the object under testing, and the construction of test stubs is a major overhead in determining the test order of class integration. Test stub complexity SCplx is used to measure the cost of building a test stub. The lower the complexity of the test stub, the less costly the test. For two classes i and j with dependencies, if test stubs need to be constructed, the individual test stub complexity SCplx(i,j) is the weighted sum of squares of the attribute complexity $A\left(i,j\right)$ and the method complexity $M\left(i,j\right)$, as shown in Equation (19).

(19) $$SCplx(i,j)=\sqrt{W_{A}\times {\left(\bar{A(i,j)}\right)}^{2}+W_{M}\times {\left(\bar{M(i,j)}\right)}^{2}}$$

where SCplx is test stub complexity. $W_{A}$ and $W_{M}$ are the weights of attribute complexity and method complexity, and $W_{A}+W_{M}=1$. $\bar{A(i,j)}$ and $\bar{M(i,j)}$ denote the results after normalization of attribute complexity and method complexity, respectively. The normalization is performed to allow comparability between the attribute complexity and method complexity. The calculation formulas of $\bar{A(i,j)}$ and $\bar{M(i,j)}$ are shown in Equations (20) and (21).

(20) $$\bar{A(i,j)}=\frac{A(i,j)}{A{\left(i,j\right)}_{\max}-A{\left(i,j\right)}_{\min}}$$

(21) $$\bar{M(i,j)}=\frac{M(i,j)}{M{\left(i,j\right)}_{\max}-M{\left(i,j\right)}_{\min}}$$

where $A\left(i,j\right)$ is the attribute complexity and represents the total number of attribute dependencies between classes i and j. $M\left(i,j\right)$ is the method complexity, representing the total number of method dependencies between class i and class j.

For a test order O, the overall stubbing complexity is shown in Equation (22).

(22) $$OCplx(O)=\sum_{i=1,j=1}^{n}SCplx(i,j)$$

where n represents the total number of classes in the test order.

When solving the CITO generation problem using the MSSA, the quality of the sparrow is evaluated by the individual fitness function, where the sparrows represents the class integration test orders. The cost of obtaining the CITO is measured by the overall stubbing complexity. Therefore, the quality of the sparrow can be evaluated by the overall stubbing complexity. The fitness function fitness of the sparrows is represented as shown in Equation (23).

(23) $$fitness=OCplx(O)=\sum_{i=1,j=1}^{n}SCplx(i,j)$$

### 3.5. The Modified Sparrow Search Algorithm for the Class Integration Test Order Generation Model

**Figure 7:**
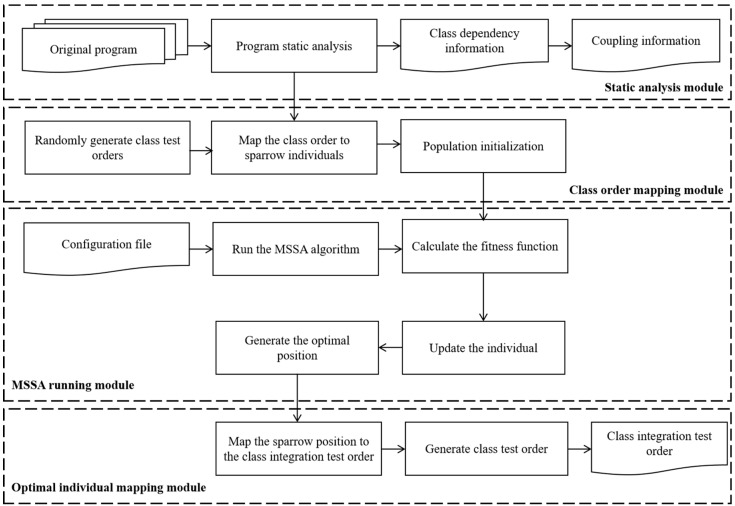
The MSSA for the CITO generation model.

The CITO generation method of the MSSA mainly includes four modules, as shown in Figure 7.

(1) The static analysis module of the software system to be tested

The system to be tested is statically analyzed to obtain the inter-class dependencies, including class name, member variable information, and member method information, and the attribute complexity $A\left(i,j\right)$ and method complexity $M\left(i,j\right)$ between classes are calculated.

(2) The class test order mapping module

All the classes contained in the source system are arranged, and the class test orders is mapped to sparrows in the sparrow population, so that each position of a sparrow corresponds to a class test order. The specific steps for mapping a class test order to the position of a sparrow individual are shown in Algorithm 4.
**Algorithm 4:** Pseudo-code for mapping the class test order to the individual position
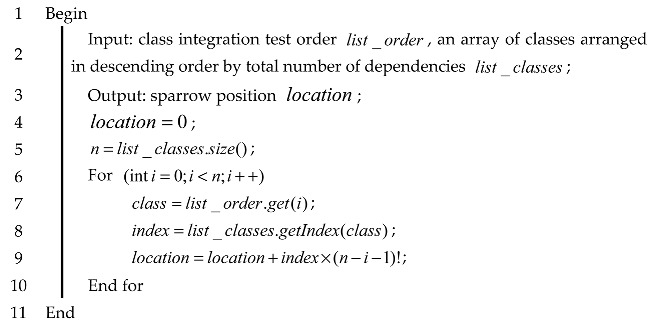


In Algorithm 4, line 4 initializes the sparrow positions and line 5 obtains the number of classes in the test order. Lines 6 through 10 map sparrow positions for n classes. The position of the i-th class in list_classes obtained in lines 7 and 8, which in turn obtains the index value of the i-th class, and line 9 finds the class information of the i-th position in a CITO. Finally, the position of a CITO in a one-dimensional space is obtained.

(3) The MSSA running module

**Figure 8:**
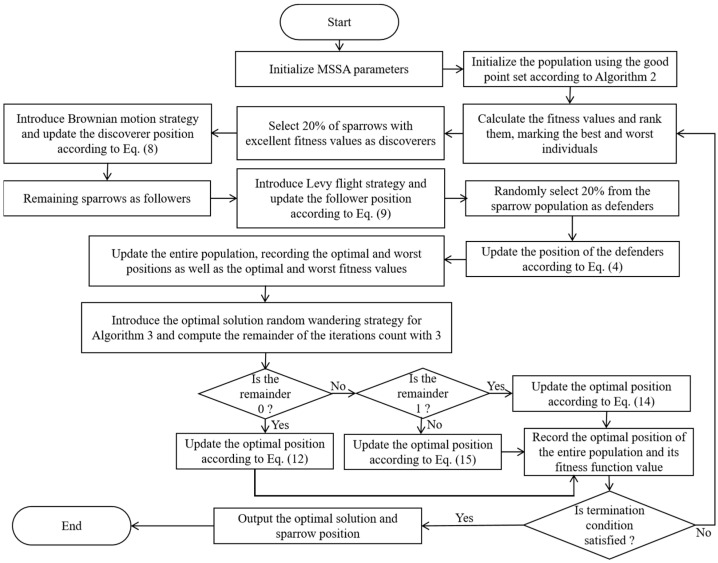
The flowchart of the MSSA.

The parameters of the MSSA are set and the sparrow swarm is initialized. The fitness function is calculated and the position of the sparrow is recorded. The optimal position of the sparrow is selected through the evolutionary update of the MSSA. The flowchart of the MSSA is shown in Figure 8, and the detailed steps of the MSSA are shown in Algorithm 5.
**Algorithm 5:** Pseudo-code of the MSSA
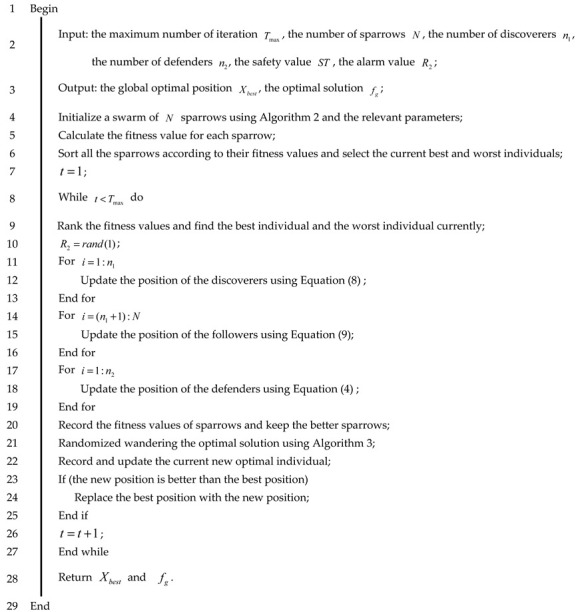

(4) The optimal sparrow mapping module

According to the mapping relationship, the optimal sparrow is mapped to its corresponding class test order, then the test order is the optimal test order.

The specific steps for mapping sparrow positions to the class integration test orders are shown in Algorithm 6.

In Algorithm 6, line 4 generates the list_classes of classes arranged in ascending order according to the sum of the number of attributes and the number of methods and counts its number. Lines 5 to 11 compute the order of integration test for the class corresponding to the sparrow position. Lines 6 and 7 obtain the position of the class in the class chain list by dividing the corresponding position information by the number of class permutations at that position. Line 8 adds the class to the liked list list_order, and line 9 deletes the class from the chain list. Line 10 obtains the position information of the remaining classes, and loops through the remaining classes to obtain the position information of the remaining classes. Lines 12 and 13 add the last class to the CITO list_order.
**Algorithm 6:** Pseudo-code for mapping the individual position to the class test order 
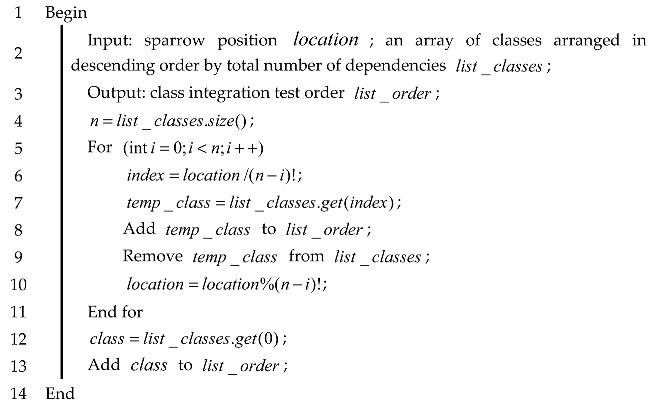


## 4. Experiments

This section describes the experimental evaluation of CITO generated by the MSSA proposed in this paper. Section 4.1 provides the information about the software system to be tested. Section 4.2 introduces the experimental environment and the parameter setting of the comparison algorithm. Section 4.3 presents the experimental results of this paper and analyzes the experimental results from the aspects of the overall stubbing complexity, attribute complexity, method complexity, convergence speed, running time, and effectiveness.

### 4.1. Experimental Subjects

To verify the performance of the proposed method on the CITO generation problem, nine Java systems are selected as experimental subjects, and the system details are shown in Table 2. Columns 1 to 6 in the table indicate that the program name, program description, number of classes included in the program, number of dependencies between classes, number of loops, and number of lines of code (LOC), respectively. The data in the table can be analyzed by Soot (http://www.sable.mcgill.ca/soot/, accessed on 2 May 2024). Elevator is a simulation system for elevator operation. SPM is a system used to simulate security patrol and monitoring. ATM is a system that simulates the automatic withdrawal of money from a bank. DEOS is an operating system kernel simulator. ANT (http://jakarta.apache.org, accessed on 2 May 2024) is a utility to update and build java applications. DEOS is an operating system kernel simulator. Email-spl (Email) is an email tool. BCEL (http://jakarta.apache.org/bcel/index.html, accessed on 2 May 2024) is a user-friendly system for analyzing, creating and manipulating Java class files. DNS (http://www.xbill.org/dnsjava/, accessed on 2 May 2024) is a Java-based domain name service system. Notepad_spl (Notepad) is a code editor. Elevator, DEOS, Email and Notepad come from the open source program systems on the SIR (software-artifact infrastructure repository, http://sir.unl.edu, accessed on 2 May 2024) web site. SPM, ATM, ANT, BCEL and DNS are derived from Briand et al. [42] and other benchmark systems. The programs under testing come from different application fields with different functionalities. The number of lines of code ranges from 934 to 6710, the number of classes ranges from 12 to 65, the number of dependencies ranges from 27 to 294, and the number of dependency loops ranges from 4 to 416,091. The variability in the complexity of these programs makes the test subjects well representative. For the larger and more complex system, it can be decomposed into several subsystems by decomposition, and the class test order for each subsystem can be found separately.

Taking the ATM system as an example, there are 21 classes in this program and the code occupies 1390 lines. The information of the classes of this system is shown in Table 3. The static analysis of the ATM system using Soot tool yields inter-class attribute dependency values and method dependency values, as shown in Table 4 and Table 5. The numbers in Table 4 and Table 5 corresponds to the numbers in Table 3. The vertical number in the table represents the source class, the horizontal number represents the target class, and the cross value between the two represents the dependency value between them. For example, (2,1) = 2 in Table 4 represents that the attribute dependency value of No. 2 Keyboard class and No. 1 Display class is 2. In Table 5, (2,1) = 1, which means that the method dependency value of No. 2 Keyboard class and No. 1 Display class is 1, etc., and a space indicates that there is no attribute and method dependency between the two classes. The space in the table indicates that there is no attribute and method dependency between the vertical and horizontal classes.

Table 6 shows the information of inter-class dependencies of the system under testing, including the maximum value, mean value and total number of dependencies of attribute dependencies and method dependencies.

### 4.2. Experiment Settings

The experimental running environment is Intel i7 CPU 2.6 GHz (Intel Corporation, Santa Clara, CA, USA), RAM 8 GB, Windows 10 operating system, miniconda3 interpreter 4.10.3, PyCharm 2021.2.3 development environment. To verify the performance of the proposed approach, the PSO algorithm [11], the cuckoo search algorithm (CS) [43], the firefly algorithm (FA) [44], the bat algorithm (BA) [45], the SCA [46], the harris hawk optimization algorithm (HHO) [47], the BSSA [48], and the MSSA are applied in the generation of the CITO. Comparative analysis is conducted from five aspects: the overall stubbing complexity, attribute complexity, method complexity, convergence speed, and running time. To ensure the effectiveness of the comparison algorithm, some parameters of the proposed algorithm in this paper are set the same as other algorithms, and the experimental parameter setting are as shown in Table 7.

### 4.3. Experimental Results and Analysis

#### 4.3.1. The Overall Stubbing Complexity

**Figure 9:**
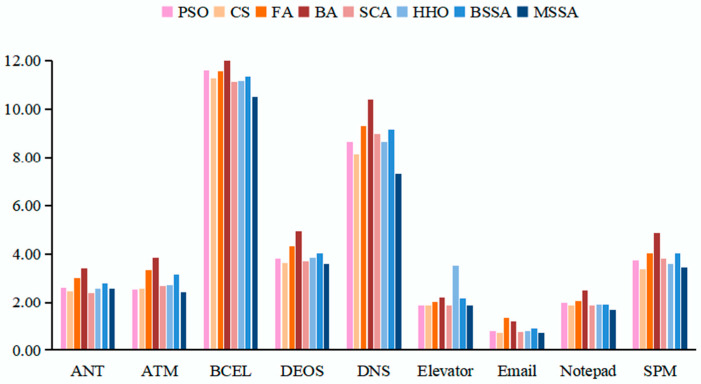
The overall stubbing complexity of the nine systems.

To guarantee experiment fairness, each algorithm is solved twenty times on its own. There can be a maximum of 200 iterations and a population size of 100. When calculating the fitness function, the values of both $W_{A}$ and $W_{M}$ are set to 0.5. The best value, worst value, mean value and standard deviation (SD) of each algorithm after running 20 times on 9 systems are recorded, and the experimental results are shown in Table 8 and Figure 9. In Table 8, the first column is the experimental subject, the second column represents the evaluation metrics of the algorithms, and columns 3 to 10 show the experimental results of various comparative algorithms. For each experimental subject, the first row is the average value of the overall stubbing complexity obtained by the algorithm, the second row is the optimal value of the overall stubbing complexity obtained by the algorithm. The third row shows the worst value of the overall complexity of test stubs obtained by the algorithm, and the fourth row shows the standard deviation of the optimization results. In Figure 9, the horizontal axis represents the individual experimental objects, i.e., ANT, ATM, BCEL, DEOS, DNS, Elevator, Email, Notepad, SPM, nine software systems. The vertical axis represents the overall complexity of the corresponding test stubs for the class test orders generated by the PSO, the CS, the FA, the BA, the SCA, the HHO, the BSSA and the MSSA. Meanwhile, Table 9 is the result of the Wilcoxon signed rank test on the data in Table 8. The symbols “+”, “−”, and “=“ indicate that when comparing two algorithms, the performance of the first algorithm is better, worse, or equal to that of the second algorithm. The “gm” represents the performance measure of the first algorithm relative to the second algorithm.

The lower the complexity of the test stub, the less expensive the test will be. As can be seen from Table 8, the overall stubbing complexity of the MSSA in generating CITO on nine test systems is lower than that of the BSSA, the PSO algorithm, the FA, the BA, and the HHO algorithm. The search performance of the MSSA is better than that of the BSSA, which indicates that the improvement strategy of this paper for the BSSA is effective, and that the optimization results of the MSSA can outperform most of the novel intelligent algorithms.

From Table 8 and Table 9, it can be seen that the MSSA proposed in this paper outperforms the other seven algorithms on nine systems. Among them, the proposed algorithm outperforms the PSO algorithm on eight systems, has no difference in performance with the PSO algorithm on the DEOS system; the MSSA is better than the CS algorithm on five systems, worse than the CS algorithm on the SPM and ANT systems, and no difference on Elevator and Notepad systems; the MSSA outperforms the FA, the BA, the HHO and the BSSA on all nine systems; the MSSA outperforms the SCA on seven systems, has worse performance than the SCA on the ANT system and has no difference in performance with the SCA on the Notepad system.

The MSSA outperforms the SCA on ATM, BCEL, DEOS, DNS, Elevator, Email, and SPM, a total of seven test systems. Among them, on ANT, the MSSA is inferior to the SCA. On Notepad, the MSSA is worse than the SCA in terms of worst value and standard deviation, but better than the SCA in terms of mean value and best value. The MSSA outperforms the CS algorithm on ATM, Email, BCEL, and DNS, a total of four test systems. On Elevator, DEOS, and Notepad systems, the MSSA and CS algorithms have the same optimization seeking performance, while the MSSA is worse than the CS algorithm on the SPM and ANT systems.

As can be seen in Figure 9, the algorithm has a lower test stub cost for obtaining CITO on the Elevator, Email and Notepad systems due to the fact that the number of classes in Elevator is 12, which is relatively small and the number of dependencies is low, and the number of dependencies in Elevator is 27, which is relatively small. The number of dependencies in Email is 61, which is relatively small. Although the Notepad system contains 65 classes, the number of classes is the largest, but its number of dependencies is 141 and the attribute and method dependencies are relatively simple, making it less costly to test stubs.

The complexity of the test stub is relatively high when the algorithm seeks for optimization in the BECL and DNS systems to obtain the CITO, this is because the BECL and DNS systems contain the largest number of classes, up to 45 and 61, respectively, and the number of dependencies of these two systems, 294 and 276, respectively. The greater number of classes increases the optimization space for the algorithm. The higher the number of dependencies, the more complex the system, the higher the overall stubbing complexity.

The overall stubbing complexity of the algorithm is in the medium when the CITO is obtained on the ATM, ANT, DEOS and SPM systems. The number of classes for SPM is 19, the number of classes for ATM is 21, and both ANT and DEOS have 25 classes. The number of dependencies for the ATM, ANT, DEOS, and SPM systems are 67, 83, 73, and 72, respectively. The number of classes and the number of dependencies for these four systems do not differ much, and all of them are in the relative middle position, so that the cost of their tests remains in the middle of the range as well.

In summary, the larger the system size, for example, on the BECL and DNS systems, the MSSA is more able to maintain superior search performance. The BSSA is prone to getting stuck in local extremes, and often the optimal solution at this time is not the actual optimal solution in the solution space, which weakens its global search ability and prevents it from escaping from local optima. The BSSA is unable to find a better CITO, while the MSSA is able to find a better CITO by introducing three optimal solution random wandering strategies along with the number of iterations. The algorithm introduces three kinds of best solution random wandering strategies in each iteration along with the number of iterations, which can search around the current position to jump out of the local optimum, avoid the phenomenon of premature convergence of the algorithm, and finally find the optimal value. The overall stubbing complexity of the MSSA for generating the CITO on nine test systems is lower than that of the BSSA, the PSO algorithm, the FA, the BA, and the HHO algorithm. The MSSA, on the one hand, uses the good point set initialization technique, which increases the quality of the initial population and distributes it uniformly over the solution space. Conversely, this is because the MSSA balances the global optimization search and local optimization capabilities by introducing BM and LF strategy in the iterative process.

#### 4.3.2. Attribute Complexity

**Figure 10:**
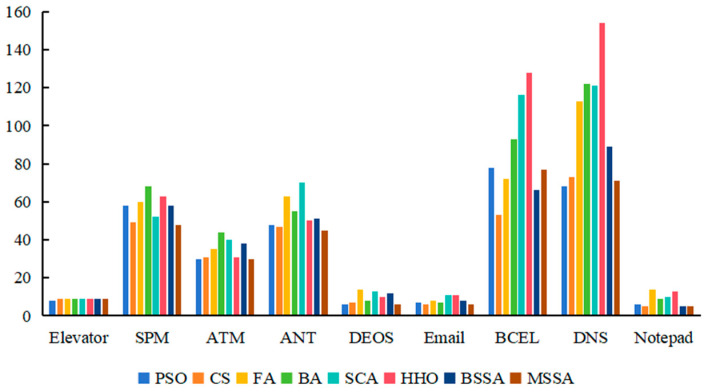
Experimental results of minimum attribute complexity.

**Figure 11:**
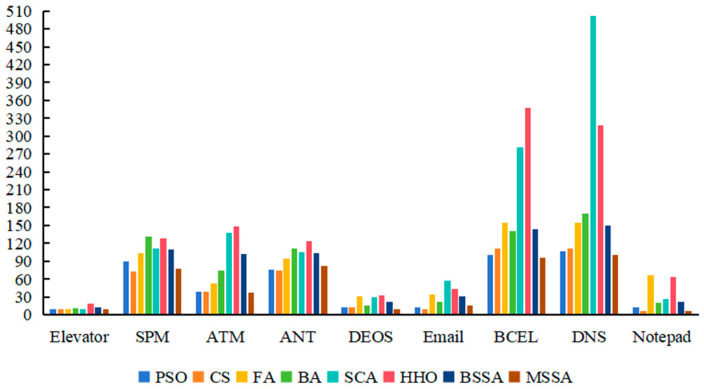
Experimental results of maximum attribute complexity.

To further analyze the complexity of algorithms to generate CITO, the attribute complexity spent by each algorithm to generate CITO is calculated, and the experimental results are shown in Table 10, Figure 10 and Figure 11. The first column in Table 10 is the system under testing, and columns 2 to 9 show the attribute complexity of the PSO, CS, the FA, the BA, the SCA, the HHO, the BSSA, and MSSA algorithms on nine experimental objects Elevator, SPM, ATM, ANT, DEOS, Email, BCEL, DNS, and Notepad. The data in each cell of the table represent the minimum to maximum range of attribute complexity obtained by executing the algorithm 20 times.

As can be seen from Table 10, Figure 10 and Figure 11, the attribute complexity of the MSSA for generating CITO is lower than that of the BSSA in nine systems, namely, Elevator, SPM, ATM, ANT, DEOS, Email, BCEL, DNS, and Notepad, which illustrates the effectiveness of the improvement strategy of the BSSA.

For the Elevator system, the attribute complexity of the MSSA for generating CITO is slightly higher than that of the PSO algorithm, consistent with the CS algorithm, and lower than that of the FA, the SCA, the BA, the BSSA, and HHO algorithms. For the Email system, the MSSA generates CITO with higher attribute complexity than that of the CS algorithm, but lower than that of the other six algorithms. The attribute complexity of generating CITO by various comparison algorithms from low to high is as follows: the CS algorithm, the MSSA, the PSO algorithm, the BA, the BSSA, the FA, the HHO algorithm, and the SCA.

For the SPM and ANT systems, the minimum value of the attribute complexity of the MSSA for generating CITO is lower than the other seven algorithms, but the maximum value of the attribute complexity for generating CITO is higher than the CS algorithm. For the BECL system, the maximum value of the attribute complexity for generating CITO using the MSSA is lower than the other seven algorithms, but the minimum value of the attribute complexity of generating CITO are higher than the CS algorithm. For the DNS system, the maximum value of attribute complexity of the MSSA is lower than the other seven algorithms, but the minimum value of attribute complexity is higher than the PSO algorithm.

For ATM, DEOS and Notepad systems, the MSSA generates class integration test orders with lowest attribute complexity compared with other algorithms. Among them, for the Notepad system with the largest number of classes, the attribute complexity of the CS algorithm to generate class integration test orders is the same as that of the MSSA, and the attribute complexity range is [5, 7]. It shows that the MSSA shows good performance in large-scale systems.

For the ATM system, the attribute complexity of the MSSA to generate the CITO is lower than that of the PSO algorithm, which is lower than that of the CS algorithm, which is lower than that of the FA, which is lower than that of the BA, which is lower than that of the BSSA, which is lower than that of the SCA, which is lower than that of the HHO algorithm. For the DEOS system, the attribute complexity of various comparison algorithms for generating CITO is, in ascending order, the MSSA, the PSO algorithm, the CS algorithm, the BA, the BSSA, the SCA, the HHO algorithm, and the FA. For the Notepad system, the order of complexity of the attributes of each algorithm for generating CITO from low to high is: the MSSA, the CS algorithm, the PSO algorithm, the BSSA, the BA, the SCA, the HHO algorithm, and the FA. It can be seen that the MSSA, the PSO algorithm and the CS algorithm spend relatively low attribute complexity in three systems ATM, DEOS and Notepad.

Overall, the MSSA has low attribute complexity and low testing cost when solving the problem of CITO generation.

#### 4.3.3. Method Complexity

**Figure 12:**
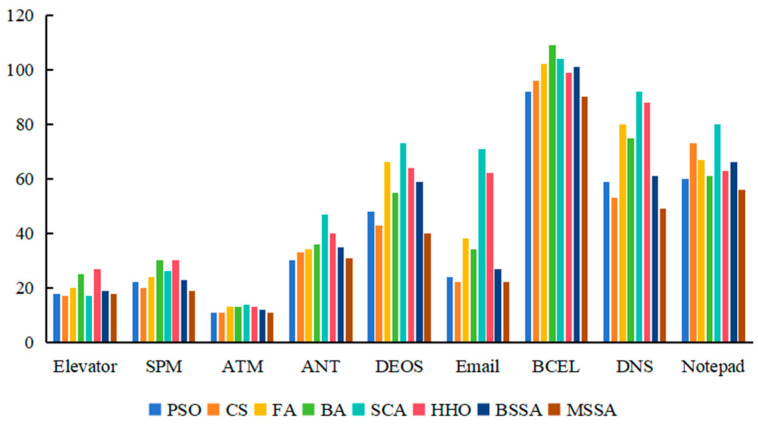
Experimental results of minimum method complexity.

**Figure 13:**
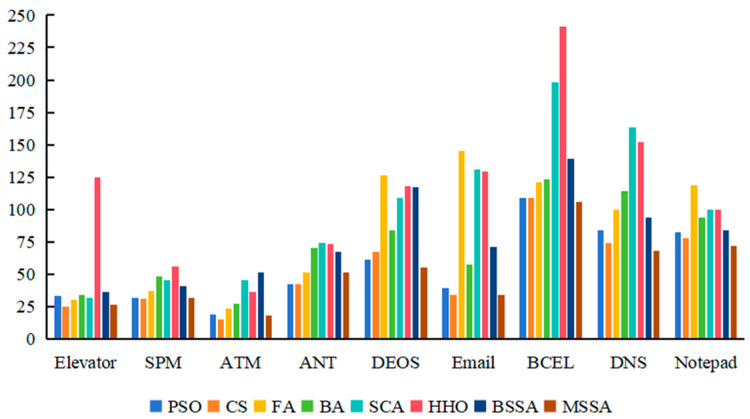
Experimental results of maximum method complexity.

To further analyze the cost of various comparison algorithms for generating CITO, the method complexity information of generating CITO by each algorithm is analyzed in the experiment, and experimental results are shown in Table 11, Figure 12 and Figure 13. In Table 11, the first column is the system under testing, and the second to ninth columns are the maximum and minimum values of the method complexity spent on generating CITO for each of the comparison algorithms running 20 times.

From Table 11, Figure 12 and Figure 13, the method complexity of the MSSA for generating CITO on the nine systems is lower than that of the BSSA, indicating the effectiveness of the algorithmic improvement strategy. For Elevator, DEOS, Email, BECL, DNS and Notepad systems, the attribute complexity of the MSSA for generating CITO is lower than the other seven algorithms. Among them, in the Email system, the CS algorithm and the BSSA generate CITO with the same method complexity, both of which are the lowest. It shows that the MSSA has the lowest method complexity for generating CITO on most of the systems and the MSSA has the best performance.

For the ATM system, the method complexity of the MSSA for generating CITO is slightly higher than that of the CS algorithm, but lower than that of the other six algorithms. For the ANT system, the method complexity of the MSSA for generating CITO is higher than that of the PSO algorithm, but lower than that of the other six algorithms. For the SPM system, the maximum value of method complexity of the MSSA to generate CITO is higher than the CS algorithm, but the minimum value of method complexity of the MSSA is lower than the other seven algorithms. It shows that the MSSA, the CS algorithm and the PSO algorithm have lower method complexity for generating CITO compared to other algorithms.

Overall, the MSSA requires low method complexity in solving the CITO generation problem, and the cost of testing is less expensive.

#### 4.3.4. Convergence Speed

**Figure 14:**
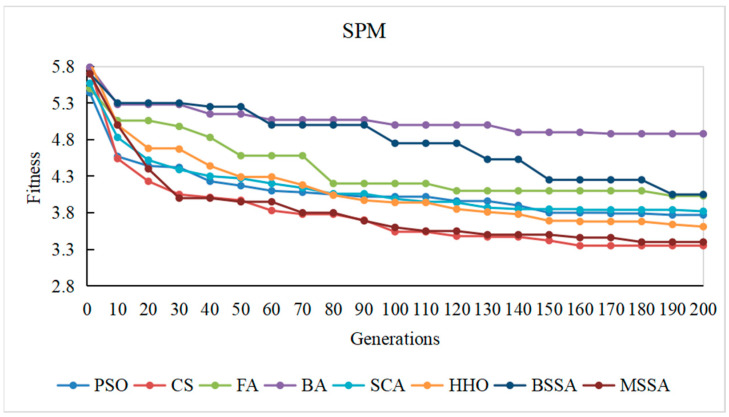
Convergence curve of the algorithm on the SPM system.

**Figure 15:**
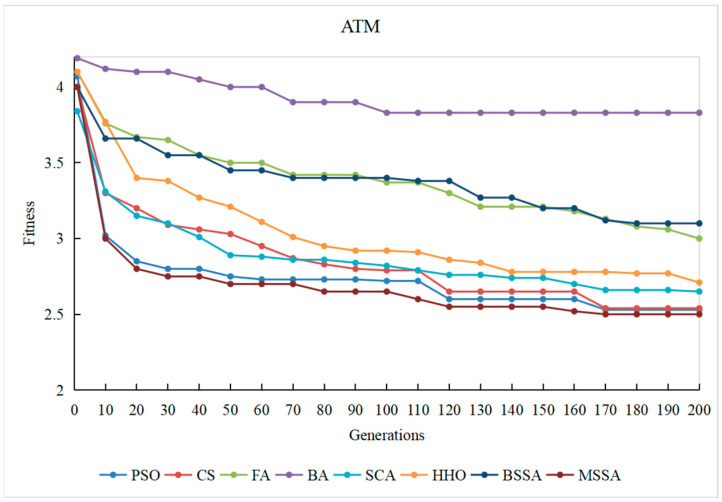
Convergence curve of the algorithm on the ATM system.

**Figure 16:**
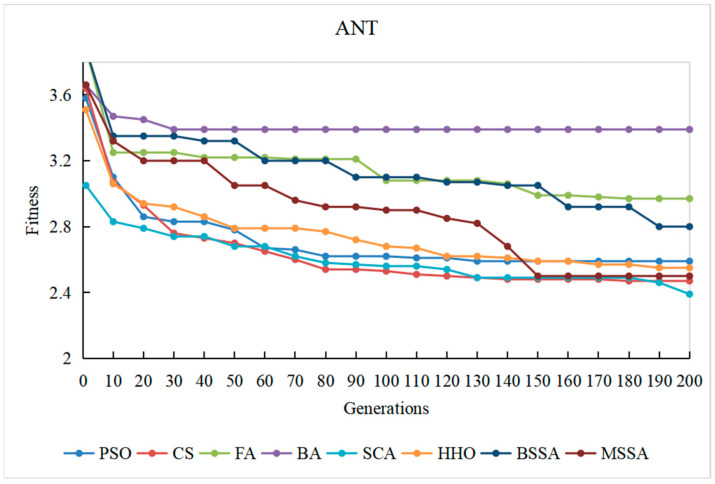
Convergence curve of the algorithm on the ANT system.

**Figure 17:**
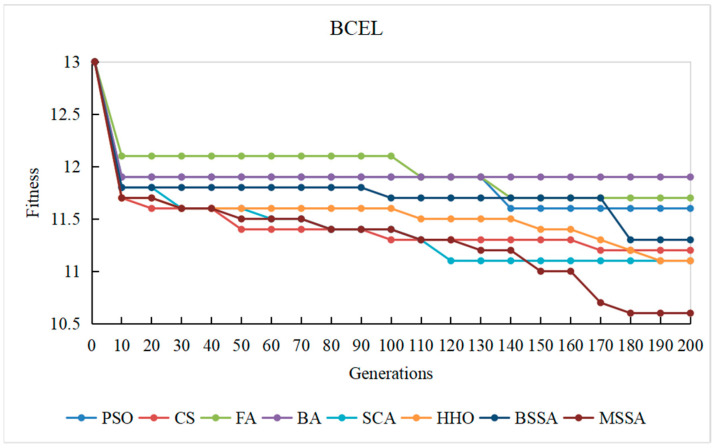
Convergence curve of the algorithm on the BCEL system.

**Figure 18:**
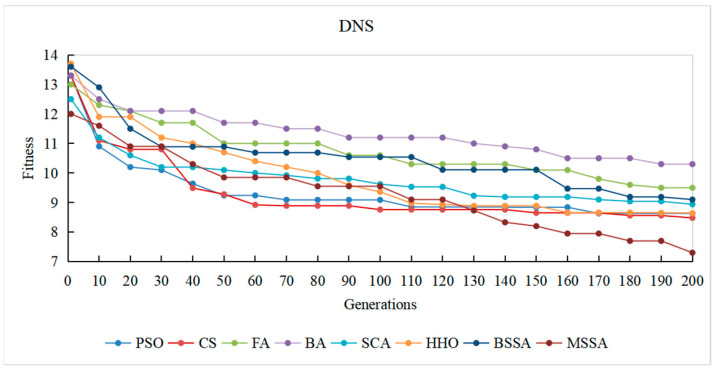
Convergence curve of the algorithm on the DNS system.

To observe the optimization process of different comparison algorithms on the problem of CITO generation in detail, the overall stubbing complexity of generating CITO is statistically varied with the number of iterations, and the convergence curves of some systems are plotted, as shown in Figure 14, Figure 15, Figure 16, Figure 17 and Figure 18. The convergence curves in Figure 14, Figure 15, Figure 16, Figure 17 and Figure 18 represent the average test stub complexity of each algorithm in 200 iterations during the algorithm run. Where, the horizontal axis represents the number of iterations, and the vertical axis is the overall test stubbing complexity consumed by each comparison algorithm to generate CITO.

As can be seen from the convergence curve in Figure 14, Figure 15, Figure 16, Figure 17 and Figure 18, the overall complexity of the test stubs decreases with the increase in the number of iterations, which verifies that the intelligent optimization algorithm can effectively solve the CITO generation problem.

From the convergence speed of the algorithm, the MSSA has a higher convergence speed and convergence accuracy than the BSSA, and it is not easy to fall into the local optimum, which indicates the effectiveness of the improvement strategy in this paper. In the early stage of algorithm iteration, the MSSA adopts the good point set strategy to initialize the swarm, which has advantageous over the random initialization strategy of the BSSA, making the overall complexity of the test stubs decrease rapidly. In the middle stage of the iteration, the optimization ability of the BSSA decreases and the convergence speed slows down, while the BM and Levy flight strategies adopted by the MSSA enable the algorithm to balance global optimization and local optimization, and the overall stubbing complexity continues to decline. In the later stage of iteration, the BSSA is already at a steady state and the algorithm falls into the local extreme value, making it difficult to reduce the overall stubbing complexity. However, the optimal solution random wandering strategy of the MSSA makes the algorithm able to escape from the local extreme value, improves the convergence rate of the algorithm, and ultimately obtains the lowest overall complexity of the test stubs.

For SPM and ATM with a relatively small system scale, the MSSA, the CS algorithm and the PSO algorithm perform well in the optimization search process. For ANT with the medium system size, the MSSA, the SCA and the PSO algorithm perform well in the optimization search process. For BCEL and DNS, which have a relatively large system scale, the MSSA, the SCA, the CS algorithm and the PSO algorithm perform well in the optimization search process. In particular, in the late iteration of the algorithm, the other algorithms converge slowly and are in a stagnant state, while the optimal solution random wandering strategy of the MSSA makes the algorithm get rid of stagnation, and the overall complexity of the test stubs continues to decrease, which also shows that the MSSA has a better chance in the optimization search process. It also shows that the MSSA has prominent advantages when the system scale is large.

In conclusion, the MSSA has high precision and stability in the process of solving the CITO generation problem, and can quickly jump out of the local optimal solution and converge to the global optimal solution in the optimization process. Therefore, the improvement method proposed can effectively improves the global search ability, avoids the occurrence of premature maturity, and prevents the algorithm from falling into the local optima during the iteration process.

#### 4.3.5. Running Time

**Figure 19:**
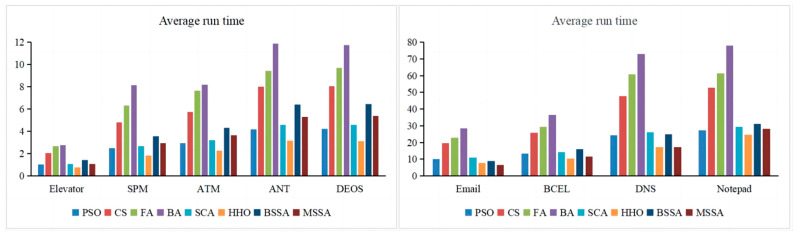
The average run time of various comparison algorithms.

For the various comparison algorithms, the time taken by them to generate the CITO is measured and the results are shown in Table 12 and Figure 19. In Table 12, the first column shows each system under testing, and the second to ninth columns are the time consumption required by each comparison algorithm to solve the problem of CITO. In Figure 19, the horizontal axis represents the experimental subjects and the vertical axis is the time consumption used by each comparison algorithm. In Table 12, the unit of time is seconds.

As can be seen from Table 12 and Figure 19, the algorithm running time increases as the system scale increases. The Elevator system has 12 classes, and the average run time required to optimize to the test order using the MSSA is 1.059 s, and the Notepad system contains 65 classes, and the average run time required to optimize to the test order using the MSSA is 28.234 s. Since classes are the basic elements that make up the test order, the running time is closely related to the number of classes. The larger the number of classes in the system under testing, the larger the size of the search space, the slower the individuals update their own positions, and the more difficult the optimization of the algorithm, thus consuming more time.

In terms of average run time, the MSSA runs in less time than the BSSA in all nine test systems, indicating that the improvement strategy of the BSSA effectively improves the performance of the algorithm. The average run time of the MSSA is less than the other six algorithms in each system, while the BA consumes the most average run time.

#### 4.3.6. Complexity Analysis

Suppose that the population size is N, the dimension of the search space is D, the maximum number of iterations is $T_{\max}$, the proportion of discoverers in the population is PD, the proportion of followers in the population is JD, and the proportion of defenders in the population is SD.

For the BSSA, the time complexity of the algorithm is $O(N\times D\times T_{\max})$.

The time complexity analysis of the MSSA is as follows. First, the time complexity of initialization with a good point set is O(N×D). Second, the time complexity of the discoverer position update based on BM is $O(N\times D\times T_{\max}\times PD)$. Then, the complexity of the position update formula for followers based on LF is $O(N\times D\times T_{\max}\times JD)$. Finally, the time complexity of the optimal solution random wandering strategy is $O(T_{\max})$. Through the above analysis, the time complexity of the MSSA is $O(N\times D)+O(N\times D\times T_{\max}\times PD)+O(N\times D\times T_{\max}\times JD)+O(T_{\max})=O(N\times D\times T_{\max})$. It can be seen that the complexity of the MSSA and the time complexity of the BSSA is on the same order of magnitude, and there is no additional consumption.

## 5. Discussion

Integration testing is a basic activity in software testing, especially in object-oriented software development. Determining the order of the classes to be integrated, i.e., the CITO generation problem, is important but computationally challenging. The research shows that the search-based algorithm can design the CITO with low test stubbing complexity, thus reducing the cost of software testing. This paper presents a CITO generation method based on the MSSA. Experimental results show the superiority of the proposed method in terms of the overall stubbing complexity, attribute complexity, method complexity, convergence speed, and running time for all systems. As with other empirical studies, there are some validity threats to the experiment. From the perspective of external validity, due to the limitation of external factors, it is impossible for any method to completely test and evaluate all the programs at any time. To further verify the feasibility and effectiveness of the MSSA and improve the persuasiveness of the experimental results, this paper selects nine experimental subjects from SIR, an authoritative repository of software engineering infrastructures. The number of classes in the selected systems ranges from 12 to 65, the number of inter-class dependencies ranges from 27 to 294, the number of inter-class loops ranges from 16 to 416,091, and the number of lines of code ranges from 934 to 6710. These systems range from different sizes and loop densities, and are representative of industry-recognized benchmark procedures, and the experimental results obtained are feasible. From the perspective of internal validity, the experimental results will be slightly different with different parameter settings of the intelligent optimization algorithm. The parameter settings of the algorithm in this paper are based on existing research and extensive experiments. To avoid the impact of randomness on the experimental results as much as possible, the results of this experiment are selected as the average of 20 times for each group of experiments, which also further proves the effectiveness of this method.

## 6. Conclusions

The CTTO problem is one of the fundamental issues in class integration testing, and the automatic generation of CITO has theoretical value and application prospects for the intelligence of software testing. The search-based approach is an important way to determine the test order for class integration testing. In this paper, a novel CITO generation method based on the MSSA is proposed, taking the overall stubbing complexity as the evaluation metric. The good point set initialization strategy makes the initial population uniformly distributed in the solution space, which provides a solid foundation for the algorithm evolution. Brownian motion and Levy flight strategy enable the algorithm to balance the exploration and exploitation ability during the evolution process, which effectively guides the evolution of the algorithm. The optimal solution random wandering strategy increases the probability of the algorithm jumping out of the local extremes, and improves the search efficiency of the algorithm. Experimental results indicate that the proposed method generates CITO with less costly test stubs.

It has been found that the MSSA has some effects on CITO generation, but there are still some shortcomings. Firstly, when dealing with systems containing more classes, the solution space of sparrows may be too large, and it can easily fall into the local optimal solution. Secondly, this paper is based on static dependency of CITO, did not consider the dynamic dependency between classes, resulting in inaccurate results, need to be studied in the future work.

In addition, the effectiveness of the proposed approach is verified in systems of different scales, there is still some distance from actually applying it to real large-scale programs in different languages to carry out the research. How to combine the method in this paper with real large-scale programs to be tested remains to be further explored in the future. In addition, we plan to conduct experimental comparisons with other multi-objective algorithms, in order to find better solutions in future work.

## Figures and Tables

**Table 1:** The stubs required by the four-class program.

Generic Stubs	Specific Stubs
B	B for A
D	D for A
	D for B

**Table 2:** Evaluation datasets.

System	Description	Classes	Dependencies	Cycles	LOC
Elevator	Classic elevator scheduling algorithm	12	27	23	934
SPM	Security patrol monitoring	19	72	1178	1198
ATM	Automated teller machine	21	67	30	1390
DEOS	Operating system kernel simulator	25	73	520	2215
ANT	A Java based build tool	25	83	654	4093
Email	Email tool	39	61	38	2276
BCEL	Byte code engineering library	45	294	416,091	3033
DNS	Domain name system	61	276	16	6710
Notepad	Code editor system	65	141	227	2419

**Table 3:** ATM system information.

Number	Class	Number	Class
0	ReceiptPrinter	11	WithdrawlTransaction
1	Display	12	DepositTransaction
2	Keyboard	13	TransferTransaction
3	CardReader	14	InquiryTransaction
4	OperatorPanel	15	GUILayout
5	EnvelopeAcceptor	16	QuestionDialog
6	CashDispenser	17	ATMMain
7	ATM	18	ATMApplet
8	Bank	19	Money
9	Session	20	Status
10	Transaction		

**Table 4:** Attribute dependence of the ATM system.

Class	0	1	2	3	4	5	6	7	8	9	10	11	12	13	14	15	16	17	18	19	20
0																2				1	
1																1					
2		2																		1	
3																	2				
4																	2			1	
5																					
6																					
7									13	9										3	
8								13													8
9								13	13												4
10								13	13	9										2	
11								13	13	9										1	2
12																				1	2
13								13	13	9										1	1
14								13	13	9										1	1
15																					
16								13	13	9											
17																				1	
18																				1	
19																					
20																					

**Table 5:** Method dependence of the ATM system.

Class	0	1	2	3	4	5	6	7	8	9	10	11	12	13	14	15	16	17	18	19	20
0																				4	
1																					
2		1																		1	
3																	2				
4													2							1	
5																					
6																					
7									1	2						1				3	
8								7													
9								7	2			2	2	2	2						
10								2	1	2											
11								4	4	2										1	
12								4	4	2											
13								3	3	2											
14								2	3	2											
15																					
16																1					
17																				1	
18																				1	
19																					
20																					

**Table 6:** Dependencies of the system under testing.

System	Attribute Dependencies			Method Dependencies			Total
	Maximum	Mean	Total	Maximum	Mean	Total	
Elevator	4	1.62	34	25	6.32	158	192
SPM	21	7.97	462	8	2.41	135	597
ATM	13	6.59	277	7	2.39	86	363
ANT	31	9.14	585	14	2.9	177	762
DEOS	4	2.04	26	15	3.28	223	249
Email	22	3.13	72	40	4.18	222	204
BCEL	8	2.52	454	4	1.55	369	823
DNS	10	4.35	766	8	1.92	328	1094
Notepad	8	1.88	102	37	1.74	181	283

**Table 7:** Various comparison algorithm parameter settings.

Algorithms	Parameters
PSO	$\begin{array}{cccc} w_{\max}=0.9 & w_{\min}=0.4 & c_{1}=2 & c_{2} \end{array}=2$
CS	$\begin{array}{ccc} P_{a}=0.02 & \alpha =0.15 & \lambda =1.5 \end{array}$
FA	$\begin{array}{ccc} \beta_{0}=2 & \gamma =1 & \alpha =0.2 \end{array}$
BA	$\begin{array}{cccc} a=0.9 & \gamma =0.7 & f_{\min}=0 & f_{\max}=2 \end{array}$
SCA	$\begin{array}{cccc} a=2 & r_{2}\in [0,2\pi ] & r_{3}\in [-2,2] & r_{4} \end{array}\in [0,1]$
HHO	$\begin{array}{ccc} E_{0}\in [-1,1] & J\in [0,2] & \beta =1.5 \end{array}$
BSSA	$\begin{array}{ccc} PD=0.2 & SD=0.2 & ST=0.8 \end{array}$
MSSA	$\begin{array}{ccc} PD=0.2 & SD=0.2 & ST=0.8 \end{array}$

**Table 8:** Comparative results of the overall stubbing complexity.

System	Statistics	Algorithm							
		PSO	CS	FA	BA	SCA	HHO	BSSA	MSSA
Elevator	Mean	1.86	1.84	2.03	2.20	1.85	3.52	2.16	1.84
	Best	1.79	1.76	1.85	1.97	1.76	1.94	1.79	1.75
	Worst	1.95	1.89	2.27	2.37	1.93	3.91	2.72	1.91
	SD	0.05	0.04	0.09	0.13	0.04	0.54	0.06	0.04
SPM	Mean	3.75	3.37	4.03	4.88	3.82	3.61	4.04	3.45
	Best	3.40	2.99	3.35	4.05	3.58	2.99	3.52	3.23
	Worst	4.16	3.80	4.99	6.37	4.19	4.08	4.56	3.89
	SD	0.22	0.27	0.46	0.62	0.19	0.30	0.33	0.23
ATM	Mean	2.53	2.54	3.30	3.83	2.65	2.69	3.12	2.43
	Best	2.17	2.29	2.26	3.02	2.32	2.27	2.31	2.19
	Worst	2.82	2.74	3.52	4.90	2.94	2.98	3.32	2.53
	SD	0.16	0.12	0.17	0.44	0.18	0.21	0.25	0.11
ANT	Mean	2.59	2.45	2.99	3.41	2.39	2.55	2.78	2.54
	Best	2.16	2.14	2.13	2.69	2.17	2.31	2.54	2.27
	Worst	2.79	2.65	2.92	4.36	2.57	2.82	3.01	2.76
	SD	0.15	0.14	0.21	0.48	0.12	0.15	0.18	0.15
DEOS	Mean	3.81	3.63	4.32	4.94	3.71	3.86	4.02	3.59
	Best	3.28	3.20	3.96	4.18	2.92	3.33	3.79	3.33
	Worst	4.18	4.02	4.84	5.39	4.05	4.46	4.27	3.86
	SD	0.28	0.24	0.62	0.36	0.33	0.28	0.41	0.29
Email	Mean	0.81	0.73	1.33	1.20	0.78	0.81	0.92	0.72
	Best	0.66	0.65	1.10	1.05	0.70	0.62	0.68	0.54
	Worst	0.89	0.81	1.85	1.43	0.91	0.92	1.03	0.76
	SD	0.06	0.05	0.20	0.11	0.06	0.07	0.09	0.04
BCEL	Mean	11.62	11.29	11.75	11.98	11.14	11.16	11.37	10.50
	Best	10.90	10.45	11.00	11.98	10.71	10.33	10.94	9.82
	Worst	11.98	11.95	13.63	11.98	11.59	11.98	11.98	11.18
	SD	0.51	0.40	0.60	0.00	0.26	0.47	0.57	0.31
DNS	Mean	8.63	8.14	9.31	10.39	8.94	8.64	9.15	7.30
	Best	7.34	7.20	6.53	8.63	7.34	6.95	6.19	5.82
	Worst	9.63	9.11	10.27	12.80	10.05	9.65	10.54	8.77
	SD	0.73	0.70	0.86	1.57	0.76	0.75	0.91	0.63
Notepad	Mean	1.98	1.85	2.07	2.50	1.85	1.90	1.93	1.68
	Best	1.83	1.77	1.76	1.90	1.80	1.77	1.72	1.57
	Worst	2.17	1.96	2.41	3.09	1.94	2.17	2.18	1.99
	SD	0.10	0.07	0.24	0.30	0.04	0.15	0.23	0.14

**Table 9:** Wilcoxon test results for algorithm simulation experiments.

Wilcoxon Rank-Sum-Test	MSSA vs. PSO	MSSA vs. CS	MSSA vs. FA	MSSA vs. BA	MSSA vs. SCA	MSSA vs. HHO	MSSA vs. BSSA
Elevator	+	=	+	+	+	+	+
SPM	+	−	+	+	+	+	+
ATM	+	+	+	+	+	+	+
ANT	+	−	+	+	−	+	+
DEOS	=	+	+	+	+	+	+
Email	+	+	+	+	+	+	+
BCEL	+	+	+	+	+	+	+
DNS	+	+	+	+	+	+	+
Notepad	+	=	+	+	=	+	+
+/−/=/gm	8/0/1/8	5/2/2/3	9/0/0/9	9/0/0/9	7/1/1/6	9/0/0/9	9/0/0/9

**Table 10:** Attribute complexity comparison results.

System	Algorithm							
	PSO	CS	FA	BA	SCA	HHO	BSSA	MSSA
Elevator	[8, 9]	[9]	[9, 10]	[9, 11]	[9, 10]	[9, 19]	[9, 13]	[9, 9]
SPM	[58, 89]	[49, 72]	[60, 103]	[68, 132]	[52, 111]	[63, 128]	[58, 110]	[48, 78]
ATM	[30, 38]	[31, 39]	[35, 52]	[44, 74]	[40, 138]	[31, 149]	[38, 102]	[30, 37]
ANT	[48, 76]	[47, 74]	[63, 95]	[55, 111]	[70, 105]	[50, 124]	[51, 103]	[45, 82]
DEOS	[6, 12]	[7, 13]	[14, 31]	[8, 15]	[13, 29]	[10, 32]	[12, 22]	[6, 10]
Email	[7, 13]	[6, 10]	[8, 34]	[7, 21]	[11, 57]	[11, 44]	[8, 31]	[6, 15]
BCEL	[78, 100]	[53, 111]	[72, 155]	[93, 140]	[116, 281]	[128, 347]	[66, 143]	[77, 96]
DNS	[68, 106]	[73, 112]	[113, 154]	[122, 170]	[121, 502]	[154, 318]	[89, 150]	[71, 101]
Notepad	[6, 12]	[5, 7]	[14, 66]	[9, 20]	[10, 26]	[13, 64]	[5, 22]	[5, 7]

**Table 11:** Method complexity comparison results.

System	Algorithm							
	PSO	CS	FA	BA	SCA	HHO	BSSA	MSSA
Elevator	[18, 33]	[17, 25]	[20, 30]	[25, 34]	[17, 32]	[27, 125]	[19, 36]	[18, 26]
SPM	[22, 32]	[20, 31]	[24, 37]	[30, 48]	[26, 45]	[30, 56]	[23, 41]	[19, 32]
ATM	[11, 19]	[11, 15]	[13, 23]	[13, 27]	[14, 45]	[13, 36]	[12, 51]	[11, 18]
ANT	[30, 42]	[33, 42]	[34, 51]	[36, 70]	[47, 74]	[40, 73]	[35, 67]	[31, 51]
DEOS	[48, 61]	[43, 67]	[66, 126]	[55, 84]	[73, 109]	[64, 118]	[59, 117]	[40, 55]
Email	[24, 39]	[22, 34]	[38, 145]	[34, 57]	[71, 131]	[62, 129]	[27, 71]	[22, 34]
BCEL	[92, 109]	[96, 109]	[102, 121]	[109, 123]	[104, 198]	[99, 241]	[101, 139]	[90, 106]
DNS	[59, 84]	[53, 74]	[80, 100]	[75, 114]	[92, 163]	[88, 152]	[61, 94]	[49, 68]
Notepad	[60, 82]	[73, 78]	[67, 119]	[61, 94]	[80, 100]	[63, 100]	[66, 84]	[56, 72]

**Table 12:** Time consumption of various comparison algorithms.

System	Algorithm							
	PSO	CS	FA	BA	SCA	HHO	BSSA	MSSA
Elevator	1.004	2.067	2.652	2.759	1.085	0.779	1.411	1.059
SPM	2.489	4.816	6.335	8.159	2.668	1.826	3.578	2.936
ATM	2.948	5.723	7.653	8.199	3.225	2.268	4.334	3.646
ANT	4.189	8.011	9.422	11.874	4.560	3.149	6.406	5.286
DEOS	4.225	8.037	9.678	11.720	4.602	3.108	6.448	5.372
Email	9.975	19.495	22.876	28.365	10.958	7.608	8.949	6.416
BCEL	13.232	25.821	29.350	36.527	14.150	10.358	15.896	11.676
DNS	24.306	47.800	60.892	72.791	26.141	17.212	25.000	17.174
Notepad	27.413	52.760	61.349	78.079	29.509	24.475	31.210	28.234
Average	9.976	19.392	23.356	28.719	10.766	7.865	11.470	9.089

## Data Availability

Data are contained within this article.

## Abbreviations

The following abbreviations are used in this manuscript:

AO	Aquila optimizer
AOA	Arithmetic optimization algorithm
CITO	Class integration test order
SSA	Sparrow search algorithm
MSSA	Modified sparrow search algorithm
GA	Genetic algorithm
PSO	Particle swarm optimization algorithm
SA	Simulated annealing algorithm
GE	Grammatical evolution
EMSSA	Enhanced multi-strategy SSA
SCA	Sine cosine algorithm
BSSA	Basic sparrow search algorithm
BM	Brownian motion
LF	Levy flight
LOC	Lines of code
CS	Cuckoo search algorithm
FA	Firefly algorithm
BA	Bat algorithm
HHO	Harris hawk optimization algorithm
SAO	Smell agent optimization
SD	Standard deviation
SIR	Software-artifact infrastructure repository
